# The Achieved Capabilities Questionnaire for Community Mental Health (ACQ‐CMH): A consumer‐based measure for the evaluation of community mental health interventions

**DOI:** 10.1002/ajcp.12599

**Published:** 2022-05-02

**Authors:** Beatrice Sacchetto, José Ornelas, Maria M. Calheiros

**Affiliations:** ^1^ Applied Psychology Research Center Capabilities & Inclusion (APPsyCI) ISPA‐Instituto Universitário (ISPA‐IU) Lisbon Portugal; ^2^ Centro de Investigação em Ciência Psicológica (CICPSI), Faculdade de Psicologia Universidade de Lisboa Lisboa Portugal

**Keywords:** capabilities approach, community mental health intervention, confirmatory factor analysis, consumer‐based measurement, evaluation

## Abstract

The capabilities approach offers a multidimensional, ecological, and agent‐centered framework that may inspire models of intervention and evaluation. A growing number of measures grounded on the capabilities approach for outcome measurement are appearing. Regarding community mental health, new consumer‐valued measures—constructed in collaboration with consumers—are here considered crucial for a transformative shift. Meanwhile, new measurements need to provide psychometric evidence to enable proper choice and application. The Achieved Capabilities Questionnaire for Community Mental Health (ACQ‐CMH) was developed in collaboration with consumers of community mental health services. It aims to assess consumers' capabilities achieved through program support. The present paper shows advancements in the measure validation through a confirmatory factor analysis within a sample of community mental health consumers (*N* = 225). Reliability and construct‐related validity were also observed. A structural solution composed of five factors and 43 items revealed a better model fit than that obtained in a previous exploratory study. Findings support the reliability, sensibility, and both convergent and discriminant validity of using the ACQ‐CMH in the evaluation of community mental health interventions. The ACQ‐CMH offers a consumer‐valued framework with specific dimensions and indicators of capabilities for use in a routine service evaluation setting.

## Highlights

- Growing number of capabilities measures to evaluate health intervention has appeared.
- Psychometric refinements and confirmatory factor analysis are fundamental to enable a proper choice and application.
- The Achieved Capabilities Questionnaire for Community Mental Health (ACQ‐CMH) is consumer‐centered once it reflects consumers' needs and goals.
- This measure may contribute to the evaluation of community‐based mental health interventions.
- The obtained dimensions and indicators may inspire a transformative mental health system.

## INTRODUCTION

### Toward a capabilities‐informed mental health system

The capabilities approach has gained visibility among diverse scientific fields in recent decades. It has emerged as an alternative framework for evaluating quality of life, considering broader dimensions of well‐being than standard utilitarian approaches (Sen, 1992), suggesting the need to look at social and political deficits that hinder access to individuals' opportunities. Capabilities are the doings and beings people choose to achieve, also defined as “combined capabilities,” comprising internal or personal characteristics and external possibilities and conditions (Nussbaum, 2011). Nussbaum (2000) proposed a list of 10 capabilities for a worthy quality of life that encompass wide‐ranging dimensions, such as emotions, affiliation, practical reason, and control over one's environment. This account has been discussed as a useful multidimensional framework for evaluating one's environment, providing for critical reflection on contextual and institutional barriers or facilities. The capabilities framework appears to share the same underlying societal and ecological perspective as community psychology, encompassing an understanding of people as social beings within interpersonal, social, institutional, and political networks (Kelly, 2006; Sen, 2009). An in‐depth multilevel analysis of one's environment and its impacts on individual opportunities is mandatory, particularly for people affected by social exclusion and discrimination. People in disadvantaged situations may suffer a capabilities deprivation, that is, a restriction of possibilities that interfere with the ability to make choices and to fully participate in society. Social policies toward equality of welfare may be understood and assessed in terms of equality of opportunity and individual preference satisfaction (Wolff, 2009). In this sense, success in addressing disability or mental health challenges depends on providing supportive environments to choose and exercise meaningful roles and activities within the community. A capabilities‐informed mental health system may search for “what enables people to thrive, not to survive […] real opportunities for exercising self‐determination and making informed life‐changing commitments” (Hopper, 2007, p. 875).

Considering community mental health responses, there are different models of intervention. Most of the “vocational‐rehabilitation” programs follow a staircase model that aims at illness management and training life skills to gradually prepare for the transition to the community (Shen & Snowden, 2014). However, research has shown that training in artificial contexts is not effective for real life in natural settings, as competences are not transferable (Corrigan & McCracken, 2005). Moreover, this model implies a perpetual professional‐led process of psychological assessment, mostly considering consumers' deficits and symptoms. A divergent model of intervention based on community psychology principles is the so‐called “intervention‐first approach” (Ornelas et al., 2019); it relies on the assumption that recovery, as a personal process, is only possible if people are involved in natural community environments and have concrete opportunities for participation (Davidson et al., 2009; Ornelas et al., 2014). For instance, regarding job placement, the model of “sheltered employment” consists of work experiences within protected environments like social firms of manufacturing or catering, while the “supported employment” model strives for work opportunities in the open labor market available to all citizens and based on individuals' preferences (Drake et al., 2012). Additionally, considering housing, the “group homes” model—provided mostly by the rehabilitative approach—is in contrast with the “independent housing” model, which promotes access to independent and permanent housing with flexible consumer‐driven support services (Tsemberis et al., 2004).

Within the Portuguese mental health system—which is the context of the current study—the majority of the national resources and consumers is still hold by the psychiatric institutions. The principal alternative is based on community mental health structures, mostly non‐profit organizations that are particularly committed to deinstitutionalization and social integration. Nevertheless, national studies have reported that most of these community services still follow institutional approaches based on vocational‐rehabilitation models of intervention, revealing a low‐recovery orientation, while programs as independent housing and supported employment indicate a high‐recovery orientation (Jorge‐Monteiro & Ornelas, 2016b).

The impacts of different intervention models on individual outcomes have to be examined to identify best practices to promote effective community integration, as well as recovery and empowerment. Empowerment, which focuses on mastery and personal power and recovery, which refers to self‐determination and meaningful connectedness within community life, is largely recognized as a pillar to orient mental health services and interventions (Corrigan, 2006; Farkas et al., 2005). These concepts are theoretically coherent and interconnected with the capabilities perspective (Davidson et al., 2009; Hopper, 2007). The capabilities approach offers further guidelines for rethinking the consumers' role, restoring their agency and control over their lives (Wallcraft & Hopper, 2015), as well as their right to choose within socially valued opportunities for integration and citizenship (Hopper, 2007).

Accordingly, new measures that consider consumers' perspectives are needed to foster their right to participate in service planning and evaluation (Wallcraft & Hopper, 2015). This is even more important because the majority of outcome measures in mental health were developed by professionals and researchers without consumers' involvement (Rose et al., 2011). The lack of participative approaches led to a definition of service outcome that may not be relevant for them or reflect their values and experiences, which is not recommended when researching quality of life (Esposito et al., 2019; Thornicroft & Tansella, 2010). In this sense, this paper offers a measure, developed in previous studies in collaboration with people with mental illness experience, which aims to assess and monitor if programs are really promoting the achievement of consumer‐valued capabilities (Sacchetto et al., 2018).

### Capabilities measures for outcome evaluation

Regarding evaluation and measurement, the capabilities framework has inspired interdisciplinary studies, comprising social and health sciences (Helter et al., 2019). A growing number of capabilities measures have appeared, in particular outcome measures (Lorgelly et al., 2015). Regarding health, the capabilities approach proposes an alternative framework for assessment of interventions considering health and non‐health effects (Mitchell et al., 2015, 2017). For mental health measurement, this is particularly pertinent as it endorses the value of non‐health outcomes such as recovery, empowerment, and social integration (Ornelas et al., 2019; Shinn, 2015).

Helter et al. (2019) presented a literature review on capabilities instruments for the evaluation of health‐related interventions. Fourteen newly developed instruments were identified, most of them with some evidence of psychometric properties, although further information about practical and theoretical characteristics of these measures is recommended to properly choose and apply them in both research and intervention. Another recent systematic review, concerning capabilities measurement in health, identified 11 questionnaires with good validity and reliability evidence (Till et al., 2021). These two literature reviews identified, so far, two capabilities instruments for mental health in intervention assessment (Helter et al., 2019; Till et al., 2021). Both are multidimensional and self‐reported measures. The first was developed based on a refinement of a capability instrument (OCAP‐18) for the assessment of public health interventions in Glasgow (Lorgelly et al., 2010, 2015). The process of refinement for mental health research (Simon et al., 2013) has been based on expert focus groups composed only of professionals, such as psychiatrists and psychologists, and on content validity and feasibility within a group of users of community treatment orders in the United Kingdom. The resulting mental health version is called the Oxford Capabilities Measure for Mental Health (OxCAP‐MH), and it has been further tested to determine its psychometric properties, such as reliability, validity, responsiveness, and feasibility (Łaszewska et al., 2019; Simon et al., 2013; Vergunst et al., 2017). More recently, it was also translated and adapted to the German context (Łaszewska et al., 2019; Simon et al., 2018). However, participatory processes within the developmental process were scarce.

The second capabilities instrument for mental health is the Achieved Capabilities Questionnaire for Community Mental Health (ACQ‐CMH) presented in this paper. It was developed following a collaborative approach with consumers of community mental health services (Sacchetto et al., 2016) to obtain a consumer‐driven research instrument for intervention assessment. For the measure's development, Nussbaum's framework inspired both the data collection and analysis, always through collaborative procedures. For data collection, focus groups with 50 consumers were organized, while data were analyzed by a steering committee composed of three consumers and two researchers to identify a pool of items and sort them according to Nussbaum's list. This participatory effort led to a questionnaire with 104 indicators organized according to Nussbaum's account of ten capabilities (Nussbaum, 2000, 2011) that reflect consumers' definitions of *beings* and *doings* they value, that is, their aspirations within their service paths. Considering that a key element of the capabilities approach is the act of choice, choosing indicators of capabilities seems not only logical but required. The first version of the measure, composed of 104 items and ordered by the 10 capabilities, was then refined in a subsequent study based on psychometric analysis (Sacchetto et al., 2018). First, content validity was assessed involving consumers beyond staff members and researchers, leading to the creation of a revised version of 98 items. Then, the 98‐item version was tested in terms of reliability and validity, including exploratory factor analysis (EFA), with a sample of community mental health consumers (*n* = 332). The factorial analysis (through PCA) and the parallel analysis test indicated a structure composed of 48 items and six dimensions with good psychometric properties (Sacchetto et al., 2018). In this sense, Nussbaum's account was revisited and adapted within the study context, obtaining specific dimensions and indicators of capabilities for people with mental illness experience. More details about the theoretical framework of the ACQ‐CMH, its relevance in community mental health, the process of development, as well as the EFA study, are available elsewhere (Sacchetto et al., 2016, 2018).

This paper aims to report advancements on a validation study of the 48‐item and six‐factor version of the ACQ‐CMH to find a robust research instrument for the evaluation of outcome measurements within community mental health interventions. Therefore, psychometric properties with a sample of community mental health consumers (*n* = 225) were examined, namely (a) factorial validity, through confirmatory factor analysis (CFA) (Kline, 2015), to check the factorial structure obtained by the previous EFA study (Sacchetto et al., 2018); (b) reliability; (c) construct‐related validity, including the capacity of the scale to discriminate subgroups of the sample regarding professional and housing status, as well as convergent and discriminant validity, where the relationships between the ACQ‐CMH and quality of life, recovery, empowerment, and distress measures were observed. Considering that the capabilities approach was originally proposed as an innovative framework to study quality of life (Nussbaum & Sen, 1993), this construct was considered essential for proving a significant relationship for convergent validity. Therefore, a strong correlation between the ACQ‐CMH and a quality of life scale was expected. Associations between recovery and empowerment scales with the ACQ‐CMH were hypothesized to further support convergent validity. Finally, distress was expected to be unrelated with the achievement of capabilities for divergent validity.

## METHODS

### Participants

Participants in this study (*n* = 225) were consumers of community mental health programs aged between 18 and 76 years (*M* = 41.03, SD = 12.43), and 44% were female. Almost all participants were Portuguese (95%) and Caucasian (90%), while 7% were from a Portuguese‐speaking African country. The majority were single (78%) and without children (79%). Three‐quarters of the sample (74%) knew their psychiatric diagnoses, and nearly half reported a schizophrenia diagnosis (45%), while one‐quarter reported a bipolar disorder (24%). Of the participants, 63% had experienced psychiatric hospitalizations, and among this group, almost a third (30%) were hospitalized once. The number of hospitalizations varied between 1 and 23 times (*M* = 3.54, SD = 3.65). Half of the participants (52%) lived with family, while 18% lived in group homes and 30% independently. The sample was divided between those who were not (55%) and those who were (45%) willing to move to another housing solution, mostly toward independent housing (71%). About half of the sample had high school education (53%) and wished to continue studying (43%). Seventy‐nine percent joined educational/training courses through the educational services of the programs they were attending. Regarding professional status, 78% were professionally inactive, either unemployed, retired, or receiving a social benefit—mostly a disability pension (36%). Fifty participants (22%) had work experience, but only 18 of them (8%) were gainfully employed, while the remaining were trainees or volunteers. Of this regular paid working group, 78% were supported by the program's employment services. On average, respondents had not been working for two decades (*M* = 19.20, SD = 13.75), although about half of the sample (55%) declared they were willing to start a new job. Utilization of service time varied from 2 months to 30 years (*M* = 6.31 years, SD = 6.44 years).

### Study context

The convenience sample was retrieved from the Portuguese community‐based mental health response, based on community programs. Most are non‐profit organizations distributed among the country, with prevalence within urban areas, and guided by psychosocial rehabilitation policies (Decree/Law no. 8/2010; legal ordinance no. 68/2017)^1^that emphasize the need for consumer independency, self‐determination, and full citizenship. In line with these policies, common goals of these community structures are recovery, empowerment, social integration, and participation.

Considering the larger social context, at the time of data gathering, the national unemployment rate was considerable (6.5%), which has a negative impact on house prices in long term. At the same time, social policies and programs to support the access to employment and housing for people in a disadvantaged situation are concentrated in urban areas of large cities and do not cover the necessity among the whole country.

### Sampling

Each of the 15 community‐based organizations that participated in the previous exploratory study (Sacchetto et al., 2018) was contacted again to ask for new participants who had not responded in the previous phase. Meanwhile, findings obtained by the previous EFA study were shared. Nine of these organizations accepted collaboration again, having integrated new consumers since the last data collection. To achieve an acceptable sample for the confirmatory analysis, eight more community programs were contacted and invited. Only two of them accepted participation. In total, 11 community programs participated in this study, nine situated in the capital region, one in the north, and one in the south of the country. In all, 23 services and programs were contacted, corresponding to all the community mental health organizations listed by the National Federation of Entities for the Rehabilitation from Mental Illness (*Federação Nacional de Entidades de Reabilitação de DoentesMentais* 2).

### Measures

The protocol for data collection was almost the same as that used in the previous exploratory study. Overall, five research instruments were included.

The ACQ‐CMH‐48 (Sacchetto et al., 2018), composed of 48 items across six dimensions was identified as the hypothesized or theoretical model to be tested here. The six dimensions of the ACQ‐CMH‐48 are optimism (13 items), affiliation (9 items), activism (8 items), practical reason (8 items), self‐sufficiency and determination (7 items), and family (3 items). The ACQ‐CMH aims at measuring consumers' capabilities achieved through the support of community mental health interventions. Items promote an individual's critical reflection concerning consumers' paths within the programs, starting with the statement “Through the program support I was able to….” and comprising a four‐point response scale, ranging from 4 (*totally achieved*) to (1) (*not achieved at all*).

The WHOQOL‐Bref (WHOQOL Group, 1998) is a 26‐item version of the quality of life scale developed by the World Health Organization. The first two items are general questions concerning health and quality of life satisfaction, and the other 24 items are distributed across four domains: Physical (seven items), psychological (six items), social relations (three items), and environment (eight items). This measure was already validated in Portugal (Vaz Serra et al., 2006). Internal consistency in the present study sample was quite good (0.84).

The K‐6 Distress Scale (Kessler et al., 2003) is a short scale composed of six items to measure nonspecific psychological distress, where a higher score indicates greater distress and symptom severity. The WHO Composite International Diagnostic Interview Advisory Committee by Yuan‐Pang Wang and colleagues performed the Portuguese translation. This measure also presented good internal consistency in the present study (0.84).

The Recovery Assessment Scale (RAS‐P) was originally developed by Corrigan et al. (2004) and already validated for the Portuguese context (Jorge‐Monteiro & Ornelas, 2016a). The 24‐item structure presents four domains—personal goals and hope (11 items), managing help needs (three items), supportive interpersonal relationships (four items), and beyond symptoms (six items). Internal consistency of the RAS in this study was almost excellent (0.89).

Finally, the Portuguese version of the Empowerment Scale, (ES‐P) (Jorge‐Monteiro & Ornelas, 2014), is a consumer‐constructed measure (Rogers et al., 1997). It consists of 25 items and a four‐factor structure, including self‐esteem and efficacy (nine items), power‐powerlessness relations (seven items), optimism and control over the future (three items), righteous anger (three items), and community activism and autonomy (six items). This measure presented a suitable internal consistency in the study (0.78).

The protocol also included socio‐demographic variables and questions concerning educational, professional, and housing achievements and goals, and mental health experiences such as diagnoses, hospitalizations, and participation in community mental health services.

### Procedures

Data were collected in paper form at the community structures after consent form assignment. Aims, procedures, and anonymity issues were also reinforced orally. A research member supported the data collection when there were comprehension or literacy issues (40.9% asked for assistance) and appealed to participants to respond to each question, especially in order for the ACQ‐CMH measure to be validated. The completion of the full protocol lasted about one hour, but not everyone was able to fulfill all the measures. Criteria for participants' eligibility were age (minimum 18 years); time within the community mental health programs (minimum two months); and a current mental illness experience.

### Data analysis

Psychometric and validity properties of the six‐factor and 48‐item version of ACQ‐CMH‐48, identified in this study as the hypothesized model, were examined. Before the confirmatory technique, pre‐analysis and screening procedures were performed to check normality, outliers, linearity, and multicollinearity. Problematic item distribution with respect to absolute values greater than two for both skewness and kurtosis were observed (Kline, 2015). Next, CFA with maximum likelihood estimation was employed to evaluate the model fit. Several commonly reported goodness of fit indices were applied to analyze the model adequacy: the chi‐square statistics (*χ* ^2^), the comparative fit index (CFI), the Tucker‐Lewis index (TLI), and the root mean square error of approximation (RMSEA). To further support comparative purposes of different models tested within the study, the expected cross‐validation index (ECVI) and the modified ECVI (MECVI) were observed, which are commonly used to compare two models estimated from the same data set, where the model with smaller values is to be preferred. The *χ* ^2^/df is recommended to be between 1.0 and 2.0 for adequate models (Hair et al., 2014). For CFI and TLI, indices below 0.85 show a poor model fit, the range between 0.85 and 0.90 indicates a mediocre but tolerable fit, while 0.90 or above is considered a good fit (West et al., 2012). The RMSEA is reported with the 90% confidence interval and a reasonable fit is indicated between 0.05 and 0.08 (Byrne, 2001; Kline, 2015). Items were expected to present significant strong loadings (>0.60) to corroborate convergent validity (Brown, 2015), where a minimum of 0.40 for acceptance was established; otherwise, its exclusion was considered.

Internal consistency of the scales and their subscales was tested with inter‐item correlations and Cronbach's *α*. To further support reliability, the composite reliability (CR) was calculated.

To pursue a thorough analysis of the hypothesized ACQ‐CMH‐48 and its construct‐related validity, discriminant and convergent validity were tested in diverse manners. First, to provide evidence for discriminant validity among the factors within the larger scale, the average variance extracted (AVE) of each factor was examined, which is commonly used to validate latent constructs (dos Santos & Cirillo, 2021). It compares the amount of variance that is captured by a factor in relation to the amount of the variance factors shared with one another. It is expected to find out that the AVE value of each factor is greater than the square of the correlation between the pairs of factors (Marôco, 2014).

Second, Spearman covariate correlations were applied to observe convergent and divergent validity of the AQC‐CMH with other related constructs and measures. Significant correlations were expected with WHOQOL‐Bref, RAS‐P, and BUES‐P, while a lack of association was expected with the K‐6 Distress Scale.

Third, to test the capacity of the ACQ‐CMH and its factors to discriminate within subgroups of the sample depending on professional and housing status, independent samples *t*‐test was applied (Calheiros et al., 2021). According to the literature based on community psychology principles presented above, differences were expected among participants who were working when compared to those who were professionally inactive, as well as among participants who lived in independent solutions versus those living in group homes or with relatives.

For all analyses, an *α* level of .05 was used to determine statistical significance. Descriptive statistics, independent samples *t*‐test, and correlation between variables were completed using SPSS, version 24, while the CFA was performed using AMOS, version 24.

## RESULTS

### Data screening

No missing data within the ACQ‐CMH to be validated were present. Sampling adequacy was confirmed by Kaiser–Meyer–Olkin (KMO; *p* = .89) and Bartlett's test of sphericity (*p* < .001). Multivariate normality was confirmed, and all items were included in the absolute value of two for both skewness and kurtosis.

### The hypothesized six‐factor model

To test the appropriateness of the six‐factor and 48‐item solution, as proposed by Sacchetto et al. (2018), CFA was performed. Factors were allowed to correlate. The hypothesized model presented a poor fit: *χ* ^2^(225) = 1609.35, *p* < .001, CFI = 0.84, TLI = 0.84, and RMSEA = 0.051, 90% CI (0.046, 0.056). All items significantly loaded on their factors. The standardized factor loadings were all above 0.40, ranging from 0.41 to 0.75, except for item 44 (“ACQ44_attend appointments regularly”), which presented a low regression weight (0.35) and was therefore excluded. Some interfactor correlations were high (range = 0.34–0.89), indicating the need for deeper examination. Thus, results on reliability, AVE, and interfactor correlations were observed (Table 1). In particular, AVE was used to provide evidence for discriminant validity among the factors within the larger scale. From the 15 possibilities of correlations between factors, four of them between the practical reason and other subscales of the ACQ‐CMH were greater than the AVE for each factor, highlighting problematic discriminant validity of this latent variable. Hence, a revised model composed of five factors, eliminating practical reason, was considered.

**Table 1:** Reliability and discriminant validity of the six‐factor model of the ACQ‐CMH

	Α	CR	1	2	3	4	5	6
Optimism (1)	0.90	0.90	**0.41**	‐	‐	‐	‐	‐
Affiliation (2)	0.83	0.83	0.78	**0.36**	‐	‐	‐	‐
Activism (3)	0.84	0.84	0.17^a^	0.21^a^	**0.41**	‐	‐	‐
Practical reason (4)	0.73	0.73	0.63	0.56	0.18^a^	**0.26**	‐	‐
Self‐sufficiency and determination (5)	0.79	0.80	0.31^a^	0.24	0.40^a^	0.44	**0.37**	‐
Family (6)	0.68	0.69	0.29^a^	0.16^a^	0.15^a^	0.29	0.11^a^	**0.44**

*Note*: Diagonal numbers in bold represent AVE. Numbers outside the diagonal represent square inter‐factor correlations.

Abbreviations: A, Cronbach's *α*; ACQ‐CMH, Achieved Capabilities Questionnaire for Community Mental Health; AVE, average variance extracted; CR, composite reliability.

^a^ It indicates evidence of discriminant validity (i.e., the square correlations between two factors are lower than the AVE of each of the correlating factors).

### The revised five‐factor model

**Figure 1:**
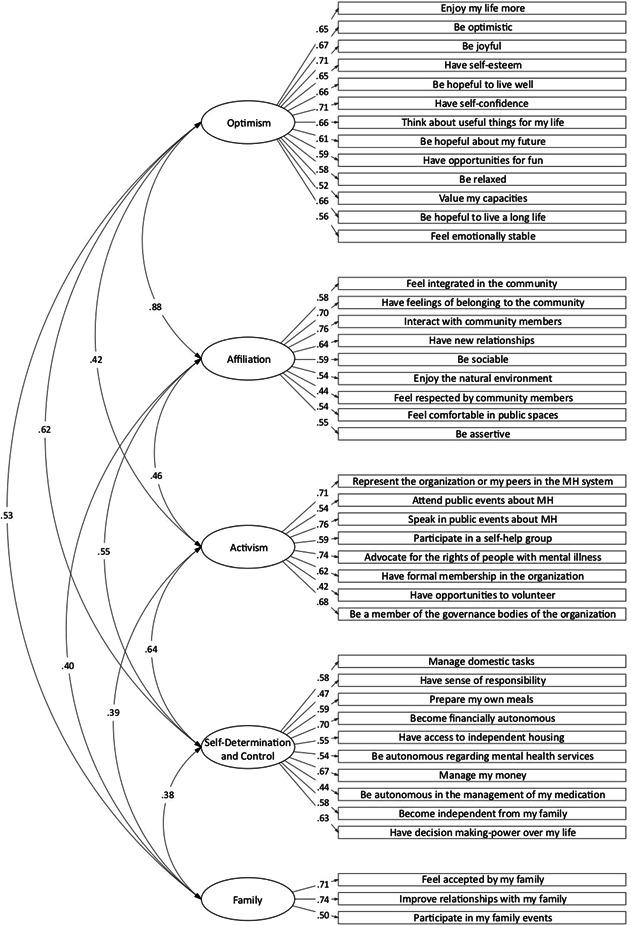
Confirmatory factor analysis of the five‐factor Achieved Capabilities Questionnaire for Community Mental Health (ACQ‐CMH)

The item within the practical reason was joined to the self‐sufficiency and determination dimension, as they presented quite close contents. Within the revised five‐factor model—called Model 2—four items concerning physical issues (e.g., “ACQ45_be aware of my physical conditions”; “ACQ4_have knowledge about healthy eating”) presented low outputs (factor loadings below 0.40) and were therefore removed. The remaining 10 items mostly report contents concerning independence, autonomy, and control. Therefore, the label of this dimension was changed into self‐determination and control. The five‐factor Model 2 presented a mediocre fit: *χ* ^2^(225) = 1310.41, *p* < .001, CFI = 0.87, TLI = 0.86, and RMSEA = 0.05, 90% CI (0.044, 0.055). However, it was better than that obtained by the six‐factor Model 1, particularly regarding the *χ* ^2^ statistic, which was significantly lower, and both the ECVI and MECVI indexes that presented smaller values for the 5‐factor Model 2. All 43 items in Model 2 significantly loaded on their factors, ranging from 0.42 to 0.76. The majority of items presented standardized factor loadings of ≥0.60, which suggests good convergent validity (Brown, 2015). However, due to a high correlation between the optimism and affiliation factors (*r* = .88), a second‐order factor for the two correlating dimensions was tested. As the model fit showed almost the same outputs than Model 2 (*χ* ^2^(225) = 1317.77, *p* < .001, CFI = 0.87, TLI = 0.86, and RMSEA = 0.05, 90% CI [0.045, 0.055]), the hypothesis of a second‐order factor was rejected. Table 2 compares the fit statistics of the two measurement models tested in this study. Considering the goodness of fit results, as well as the consistency with the theoretical framework, the five‐factor Model 2 was identified as the better one for use with a psychometric study. Figure 1 displays the five‐factor standardized Model 2 solution for the ACQ‐CMH, the factor loadings of the items on each factor, and the correlations between factors. Model 2 is composed of a total of 43 items distributed throughout five dimensions, namely: optimism (13 items); affiliation (nine items); activism (eight items); self‐determination and control (10 items); and family (three items).

**Table 2:** Goodness‐of‐fit statistics for the measurement models

	*χ* ^2^	df	*χ* ^2^/df	CFI	TLI	RMSEA (90% CI)	ECVI	MECVI
Six‐factor Model 1	1609.35	1014	1.59	0.84	0.84	0.051 (0.046–0.056)	8.2	8.48
Five‐factor Model 2	1310.41	845	1.55	0.87	0.86	0.050 (0.044–0.055)	6.75	6.69

Abbreviations: CFI, comparative fit index; ECVI, expected cross‐validation index; MECVI, modified ECVI; RMSE, root mean square error of approximation; TLI, Tucker‐Lewis index; χ^2^, chi‐square statistics.

Cronbach's *α* and CR results of the five‐factor Model 2 are presented in Table 3. The overall scale showed high internal consistency (0.94) and CR (0.96). Subscales also presented satisfactory results, ranging from .90 to .68 for *α* and 0.90 to 0.69 for CR. Corrected item‐total correlations ranged from 0.31 to 0.69. Family revealed less reliability (*α* = .68; CR = 0.69), although it was still tolerable. The self‐determination and control (*α* = .83; CR = 0.83) of Model 2 presented better results than the practical reason (*α* = .73; CR = 0.73) and self‐sufficiency and determination (*α* = .79; CR = 0.80) of Model 1 (Tables 1 and 3).

**Table 3:** Reliability of the five‐factor model of the ACQ‐CMH

Scale	No of items	*α*	CR
ACQ‐CMH overall	43	.94	0.96
ACQ‐CMH_optimism	13	.90	0.90
ACQ‐CMH_affiliation	9	.83	0.83
ACQ‐CMH_activism	8	.84	0.84
ACQ‐CMH_self‐determination and control	10	.83	0.83
ACQ‐CMH_family	3	.68	0.69

Abbreviations: ACQ‐CMH, Achieved Capabilities Questionnaire for Community Mental Health; CR, composite reliability.

To support construct‐related validity, convergent and divergent validity was tested through bivariate correlations between the total scores of the ACQ‐CMH, its five subscales (considering Model 2), and the other protocol measures. The results are shown in Table 4.

**Table 4:** Pearson correlations of the five‐factor ACQ‐CMH with its subscales, WHOQOL‐Bref, RAS‐P, BUES‐P, K6

Scale	ACQ‐CMH	ACQ1	ACQ2	ACQ3	ACQ4	ACQ5
ACQ‐CMH	‐	‐	‐	‐	‐	‐
ACQ1. Optimism	0.86	‐	‐	‐	‐	‐
ACQ2. Affiliation	0.81	0.78	‐	‐	‐	‐
ACQ3. Activism	0.69	0.37	0.38	‐	‐	‐
ACQ4. Self‐determination and control	0.79	0.53	0.48	0.54	‐	‐
ACQ5. Family	0.52	0.43	0.34	0.28	0.29	‐
WHOQOL‐Bref	0.51	0.55	0.43	0.23	0.33	0.28
RAS‐P	0.41	0.48	0.36	0.17*	0.25	0.19*
BUES‐P	0.32	0.35	0.22	0.22	0.21	0.12**
K6	−0.19	−0.27	−0.17*	−0.06**	−0.11**	−0.02**

*Note*: Correlations are significant at *p* < .01 level, with the exceptions given in the footnotes.

Abbreviations: ACQ‐CMH, Achieved Capabilities Questionnaire for Community Mental Health; BUES‐P, Portuguese version of the Empowerment Scale; K6, Distress Scale; RAS‐P, Portuguese version of the Recovery Assessment Scale; WHOQOL‐Bref, World Health Organization Quality of Life Bref.

* Significant at *p* < .05 level.

^**^ Not significant at *p* < .05.

Strong estimate correlations were obtained among the overall ACQ‐CMH and its subscales (range = 0.52–0.86). Convergent validity was supported by all the measures encompassed in the study protocol for this purpose, that is, with RAS‐P (*r*(208) = .41, *p* < .01), BUES‐P (*r*(195) = .32, *p* < .01), and WHOQOL‐Bref (*r*(171) = .51, *p* < .01). Divergent validity was also confirmed by a lack of significant correlation with the Distress Scale (*r*(197) = −.17, *p* < .05). Regarding the new latent variable labeled self‐determination and control, it showed a strong connection to the overall ACQ‐CMH (*r*(225) = .79, *p* < .01) and significant positive correlations with both the RAS (*r*(208) = .25, *p* < .01) and ES (*r*(195) = .21, *p* < .01). Considering correlations between the other dimensions of the ACQ‐CMH and the recovery, empowerment, and quality of life subscales, some relevant results are here reported. A significant strong correlation was evident between the Optimism factor and the RAS subscale of personal goals and hope (*r*(210) = .48, *p* < .01). However, the RAS Supportive Interpersonal Relationships subscale and the ACQ‐CMH Family dimension were not highly related (*r*(209) = .16, *p* < .05), corroborating results of the exploratory study (Sacchetto et al., 2018). Regarding ES, the strongest correlations were observed between the subscales self‐esteem and efficacy (*r*(199) = .25, *p* < .01) and optimism and control over the future (*r*(198) = .31, *p* < .01) with the optimism dimension of the ACQ‐CMH. Finally, correlations with the quality of life scale also showed an adequate convergent validity; in particular, the psychological health subscale was strongly associated with optimism (*r* (194) = .49, *p* < .01] and the social relationships subscale with the ACQ‐CMH affiliation (*r*(192) = .28, *p* < .01).

To further support construct‐related validity of the five‐factor ACQ‐CMH, the differences in professional and housing statuses in ACQ‐CMH factors were observed. Regarding professional status (working vs. professionally inactive), an independent samples *t*‐test with the whole sample showed significant differences in self‐determination and control (*t*(223) = 4.74, *p* < .01) and in optimism (*t*(223) = 2.25, *p* < .05). Specifically, findings revealed that participants actively engaged in professional activities (employed, trainee, or volunteer) were rated on the ACQ‐CMH as having more self‐determination and control (*M* = 3.24, SD = 0.58) than participants without a professional commitment (*M* = 2.75, SD = 0.67). Regarding housing status (independent living vs. living with family or in group homes), significant differences were observed for self‐determination and control (*t*(223) = 4.8, *p* < .01], as well as for activism (*t*(223) = 2.01, *p* < .01). Accordingly, people who lived on their own were rated at higher levels of self‐determination and control (*M* = 3.17, SD = 0.62) and activism (*M* = 2.22, SD = 0.83) than those living with relatives or in group homes (*M* = 1.99, SD = 0.66; *M* = 2.75, SD = 0.79). Thus, the scale showed sensibility among differences of the population in study, evidencing construct validity.

## DISCUSSION

Given the scarcity of measures that consider consumers' meanings of outcomes in mental health, this study aimed at presenting a consumer‐valued research instrument, inspired by Nussbaum's capabilities approach, for the evaluation of community mental health interventions. Advancements on the validation study of the ACQ‐CMH are reported, to provide evidence for a proper choice and application in both research and intervention. Best practices in the validation of new measures recommend an evaluation through CFA, to validate whether the hypothetical structure is adequate (Boateng et al., 2018). Therefore, the appropriateness of the hypothesized model obtained by the EFA study was tested, revealing a poor fit within the present sample of community mental health consumers (*n* = 225). Literature warns that follow‐up studies often fail to confirm the model structures obtained by previous explorative studies through EFA (Van Prooijen & Van Der Kloot, 2001). Since EFA is a data‐driven technique, fewer restrictions within the procedures than those for CFA are required, for instance, regarding the number of factors to retain. Although the parallel analysis test (O'Connor, 2000) was applied in the EFA study for factor retention (Sacchetto et al., 2018), particular attention was paid to the adequacy of the six latent variables of the hypothesized model (labeled Model 1) in this study. Therefore, an in‐depth analysis of factors' discriminant validity was carried out and scarce results were found for the practical reason dimension which, in fact, displayed contents quite close to those of the self‐sufficiency and determination factor. This similarity was already observed within the EFA study (Sacchetto et al., 2018). The five‐factor solution (identified as Model 2) tested in the present study proposed a unique factor called self‐determination and control composed of 10 items with a standard loading above 0.40. Contents of these indicators consistently address individual autonomy and independence regarding housing, financial issues, mental health services, and medication, as well as with regard to relatives (e.g., “ACQ34_have access to independent housing”; “ACQ21_become financially autonomous”; “ACQ17_be autonomous regarding mental health services”; “ACQ27_become independent from my family”). Self‐determination and control concerning one's life and environment are clearly evoked within these items. Moreover, item 28 (“ACQ28_have decision‐making power over my life”) directly refers to one's power and decision‐making. Theoretical coherence with recovery and empowerment is also noticeable. People with mental health issues historically suffer a lack of self‐determination, power, and control over their lives, as well as within the mental health system (Nelson et al., 2017). Accordingly, the latent factor of self‐determination and control is particularly relevant for the group in the study and inspiring for service providers; supporting consumers in achieving their full capacity of self‐determination and control over their life domains, contributing to their empowerment and recovery processes, and should be a priority in terms of service outcomes.

The five‐factor Model 2 was identified as the better model solution, considering both psychometric and theoretical criteria. However, the correlation between Optimism and Affiliation factors was high. This output may be interpreted through literature support. In‐depth research about the meaning of quality of life for people with mental health challenges reveals dimensions such as feelings of hope, belonging, and relationships (Connell et al., 2012, 2014; Gee et al., 2003). Relationships and sense of belonging are related to the experience of connectedness and of feeling accepted, which are comprised of social support, supportive relationships, and community integration. These elements are quite close to the affiliation factor (e.g., “ACQ3_feel respected by community members”; “ACQ16_have feelings of belonging to the community”; “ACQ8_feel integrated in the community”; “ACQ29_have new relationships”). While hopefulness is linked to having goals and aspirations, including coping strategies, the abilities to make plans, and to have purposes for the future (Gee et al., 2003) and converges with optimism (e.g., “ACQ31_think about useful things for my life”; “ACQ30_be optimistic”; “ACQ35_enjoy my life more”; “ACQ2_be hopeful about my future”). Therefore, a conceptual distinction for optimism and affiliation is coherent. Nussbaum states that *affiliation* and *practical reason* permeate all other dimensions of her list (Nussbaum, 2000, 2011). Based on the results of our study, we observed that Optimism goes beyond affiliation and self‐determination and control as the most relevant capabilities for people with mental illness experience.

Beyond adjusting the dimension of self‐determination and control to identify a better model solution, findings of reliability and construct‐related validity confirmed the adequacy of the dimensions and indicators of the five‐factor ACQ‐CMH, as well as its relevance for the community mental health context. Findings of convergent validity corroborate the supposed theoretical links of the ACQ‐CMH with WHOQOL‐Bref, RAS, and ES measures. The link with empowerment was hypothesized based on findings of the exploratory principal component analysis (Sacchetto et al., 2018). Therefore, an ES was added in the present study, endorsing convergent validity. Actually, the ES subscales of self‐esteem and efficacy and optimism and control over the future showed a strong association with the ACQ‐CMH optimism factor. In fact, optimism covers items concerning self‐esteem, self‐confidence, and hopefulness about the future (“ACQ48_have self‐esteem”; “ACQ40_have self‐confidence”; “ACQ14_value my capacities”; “ACQ2_be hopeful about my future”). The low association between RAS supportive interpersonal relationships and ACQ‐CMH family was already observed in the exploratory study (Sacchetto et al., 2018) and reaffirms the existence of scarce support within the family. At the same time, an item of Self‐Determination and Control indicates the need for the group in the study to become independent from their families (“ACQ27_become independent from my family”), revealing a complex and urgent topic to be addressed, considering that half of the sample still lives with the family.

Moreover, the significant differences between groups in different conditions regarding their professional and housing statuses revealed an adequate sensibility and construct validity of the ACQ‐CMH factors. Higher rates of functional capabilities were found for people who were actively engaged in work activities and who were living independently. The exploratory study had already suggested individual independence in terms of employment and housing as crucial vehicles for the achievement of capabilities (Sacchetto et al., 2018). These findings meet the measure's purpose and the theoretical basis of the capabilities framework, which advocates for socially valued roles and activities as well as agency and self‐determination.

### Implications for practice and research

By advancing psychometric analysis for the ACQ‐CMH, we aimed to provide a new evaluative measure for community mental health programs that may assess its results (and plan its intervention) based on the achievement of consumers' capabilities. This is particularly required considering that even though there have been advancements in the deinstitutionalization process, the psychiatric and institutional perspectives still dominate the psychosocial initiatives within the community mental health system, which is still focused on illness‐centered interventions and evaluation (Jorge‐Monteiro & Ornelas, 2016b). This study calls for a social model of intervention to switch from pre‐established, professional‐led, and deficit‐centered interventions to consumer‐centered and capabilities‐oriented alternatives. Accordingly, evaluation should reflect consumer‐valued gains and goals.

The ACQ‐CMH offers dimensions that may be embedded in policy and program guidelines. It claims for the promotion of complex capabilities, as the exercise of self‐determination, control, hopefulness and affiliation, beyond primary goods, such as material and financial necessities. Optimism and affiliation indicate the need for a long‐term perspective of optimism and hopefulness, through activities and roles that promote social commitments, membership, and sense of belonging to the community. Individual feelings of acceptance and social respect, beyond self‐esteem and self‐confidence, seem also to contribute to consumers' quality of life. Moreover, leisure and connection with nature are consumer‐valued elements for well‐being. While activism and self‐determination and control suggest the need to provide formal roles of governance to achieve consumer participation in service planning, delivery, and evaluation, for instance, providing opportunities for board membership. Finally, consumer‐led and peer‐support facilities, as well as advocacy initiatives, seem to have a decisive role in the promotion of consumer capabilities.

The ACQ‐CMH aims at supporting service outcomes evaluation, measuring consumers' achievements obtained through their participation in community mental health services. We expect that repeated measurements within time intervals in services' routine practices may help mental health professionals to look at consumers' gains in capabilities. Consequently, a more efficient intervention may be planned to increase individual potential, improving both the services' response and consumers' quality of life.

### Limitations and further studies

The study presents some limitations. For the data collection, a large sample size, as expected for the confirmatory analysis, was not achieved. However, all consumers available at the time participated in the collection of the data. In‐depth analysis of the factorial structure was conducted, including CFA, and other psychometric characteristics were reported, including reliability, sensibility, and construct‐related validity. Follow‐up studies may elaborate on the validation process for the measure of robustness. In particular, other psychometric properties may be analyzed, such as responsiveness, predictive validity, and sensitivity to change. Repeated data collection may be applied to observe variability of results depending on time differences (for instance, baseline, and 6‐month follow‐up). The ACQ‐CMH seeks to capture if programs are helping people to accomplish what they value, asking about achieved functional capabilities. Although the achievement of functionings is a complex and multicasual process, composed of co‐animating elements, there is an urgent need to evaluate if consumers are feeling satisfied in how services are attending to their needs' and preferences. The innovative element of the ACQ‐CMH is that the evaluation process is based on consumer‐valued indicators to assess programs' supports and services. However, a multimethod study embedding qualitative approaches may support a major comprehension of the efficacy of care interventions in promoting freedoms to be and to do, by investigating consumers' perceptions of individual possibilities and potential opportunities.

Future studies may also examine the effect of programs such as independent housing and supported employment. The findings may serve as recommendations for effective interventions for the promotion of capabilities.

Finally, pre‐established international partnerships were retained. The ACQ‐CMH was already translated into the Italian language through a cross‐cultural adaptation process (Beaton et al., 2000), and data collection is ongoing (*n* = 98) for a national validation of the Italian version of the ACQ‐CMH. Meanwhile, a partnership with a North‐American research group is revising the English version of the measure to start transnational research. Hence, we expect to obtain a well‐established and multilanguage questionnaire that facilitates comparative international data, contributing to a transformative change in community mental health.

## CONFLICTS OF INTEREST

The authors declare no conflicts of interest.

## Footnotes

1 The DL no 8/2010 is available at https://dre.pt/application/conteudo/616776, and the legal ordinance no 68/2017 is available at https://dre.pt/application/conteudo/106471884.

2
https://www.fnerdm.pt/.
